# Alismatis Rhizoma Triterpenes Alleviate High-Fat Diet-Induced Insulin Resistance in Skeletal Muscle of Mice

**DOI:** 10.1155/2021/8857687

**Published:** 2021-02-02

**Authors:** Xiao-Kang Jia, Jin-Fang Huang, Xiao-Qiang Huang, Xiao-Yan Li, Ming-Qing Huang, Huai-Chang Zhu, Gao-Pan Li, Meng-Liu Lan, Zhi-Wen Yu, Wen Xu, Shui-Sheng Wu

**Affiliations:** ^1^College of Pharmacy, Fujian University of Traditional Chinese Medicine, Fuzhou 350122, China; ^2^Academy of Integrative Medicine, Fujian University of Traditional Chinese Medicine, Fuzhou 350122, China; ^3^Centre of Biomedical Research & Development, Fujian University of Traditional Chinese Medicine, Fuzhou 350122, China; ^4^Medical School, Huanghe Science & Technology College, Zhengzhou 450063, China

## Abstract

Alismatis rhizoma (AR), which is the dried rhizome of *Alisma orientale* (Sam.) Juz. (Alismataceae), is an important component of many famous Chinese formulas for hypoglycemic. This study aimed to evaluate the insulin resistance (IR) alleviating effects of AR triterpenes (ART) and ART component compatibility (ARTC, the mixture of 16-oxo-alisol A, 16-oxo-alisol A 23-acetate, 16-oxo-alisol A 24-acetate, alisol C, alisol C 23-acetate, alisol L, alisol A, alisol A 23-acetate, alisol A 24-acetate, alisol L 23-acetate, alisol B, alisol B 23-acetate, 11-deoxy-alisol B and 11-deoxy-alisol B 23-acetate) in high-fat diet-induced IR mice and plamitate-treated IR C2C12 cells, respectively. A dose of 200 mg/kg of ART was orally administered to IR mice, and different doses (25, 50, and 100 *μ*g/ml) of ARTC groups were treated to IR C2C12 cells. IPGTT, IPITT, body weight, Hb1AC, FFA, TNF-*α*, MCP-1, and IR-associated gene expression (p-AMPK, p-IRS-1, PI3K, p-AKT, p-JNK, and GLUT4) were measured in IR mice. Glucose uptake, TNF-*α*, MCP-1, and IR-associated gene expression were also measured in IR C2C12 cells. Results showed that ART alleviated high-fat diet-induced IR in the skeletal muscle of mice, and this finding was further validated by ARTC. This study demonstrated that ART presented a notable IR alleviating effect by regulating IR-associated gene expression, and triterpenes were the material basis for the IR alleviating activity of AR.

## 1. Introduction

In recent years, increasing research attention has been paid to novel, effective antidiabetes agents derived from natural sources [1]. Alismatis rhizoma (AR) is the tuber of *Alisma orientale* (Sam.) Juz. (Alismataceae), which is an aquatic plant that has been cultivated mainly in oriental countries (e.g., China, Japan, and Korea) but is also widely distributed in other areas (e.g., North America and Europe). AR has various activities, such as diuretic, hyperlipidemic, inflammatory, antitumoral, and damp-heat clearing activities [2–8]. AR is traditional folk medicine that has long been used to promote health and longevity (Sheng Nong's herbal classic) for more than several thousand years. AR is currently included in the Pharmacopoeia of China not only as a diuretic but also as a representative hypoglycemic and hypolipidemic traditional Chinese herbal medicine. AR is an important component of many famous Chinese formulas for hypoglycemic from Febrile and Miscellaneous Disease (Shang Han Lun in Chinese) or Synopsis of Golden Chamber, including Zexie docotion, Ba wei shen qi wan, and Liu wei di huang wan [5, 9, 10], for usage as hypoglycemic because of their low toxicity, high effectiveness, and minimal side effects. Our previous studies reported the hypoglycemic activity of the ethanol extract of AR [11, 12]. Fourteen terpenoids were isolated from the ethanol extract of AR triterpenes (ART), which could promote glucose uptake in 3T3-L1 cells [13]. Meanwhile, the potential effect of ART on glucose metabolism, especially insulin resistance (IR), has not been investigated.

Type 2 diabetes (T2MD) comprises 90% of the total number of diabetes cases around the world. IR is an early metabolic abnormality in the progression of T2DM [14]. IR, which is characterized by low efficacy of insulin-induced glucose uptake [15], is caused by the dysregulated insulin-related signaling and glucose metabolism defects, such as impaired glucose transportation, phosphorylation, oxidation, and glycogen synthesis [16–18]. Apart from such well-recognized factors, low-grade inflammation is also an important contributing factor to IR [19]. Proinflammatory cytokines, such as tumor necrosis factor alpha (TNF-*α*) and monocyte chemotactic protein-1 (MCP-1), aggravate IR in patients [20, 21]. As a result, alleviation and drug intervention of IR are major tasks to lessen the risk of T2DM.

Skeletal muscles are the main target organs for insulin-mediated glucose uptake, metabolism, and utilization and is also the earliest and most important site of IR [22, 23]. In skeletal muscle, insulin biding with insulin receptor (IRS-1) usually leads to four-stage glucose transporter 4 (GLUT 4) translocation in a complex process through IRS-1/phosphatidylinositol 3-kinase (PI3K)/AKT-GLUT4 pathway [24, 25]; this pathway is responsible for insulin-induced glucose uptake. However, glucose homeostasis is broken in IR cases [14, 26]. AMP-activated protein kinase (AMPK) is a key regulator of energy homeostasis and ameliorates inflammation through inhibition of nuclear factor-*κB* (NF *κB*) activity [27]. These kinases, such as c-Jun N-terminal kinase (JNK), are activated in high-fat diet-induced or saturated fatty acid-induced IR, which catalyzes the phosphorylation of serine residues in IRS-1; this condition ultimately results in decreased phosphorylation of IRS-1 tyrosine residues and the activity of insulin-activated downstream signaling pathways [28–38]. Currently, the therapies for IR focus on reconstructing glycemia levels in subjects by following the recommended lifestyle [30]. Several insulin sensitization agents are commonly used, including thiazolidinediones (TZDs) (e.g., rosiglitazone and pioglitazone) and biguanides (e.g., metformin), which could improve IR and increase the utilization efficacy of circulating insulin; these improvements ultimately decrease the blood glucose [31]. However, TZD drugs have an increased risk of fracture and bladder cancer, especially for cardiovascular aspects [32–34]. More safe and efficient pharmacological intervention is required due to the limitation.

**Figure 1:**
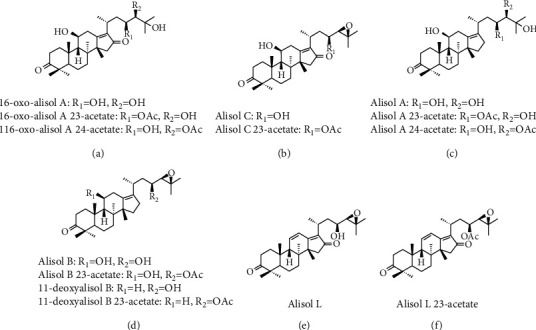
Chemical structures of the 14 compounds used for ARTC studies.

Thus, this study primarily aimed to evaluate the effect of ART and in the treatment of IR mice through body weight, glucose tolerance, hemoglobin A1c (HbA1c), Free Fatty Acid (FFA), inflammatory factors (e.g., TNF-*α* and MCP-1), AMPK/JNK, and IRS-1/PI3K/AKT/GLUT4 signaling pathway and further validated by ART component compatibility (ARTC, including 16-oxo-alisol A, 16-oxo-alisol A 23-acetate, 16-oxo-alisol A 24-acetate, alisol C, alisol C 23-acetate, alisol L, alisol A, alisol A 23-acetate, alisol A 24-acetate, alisol L 23-acetate, alisol B, alisol B 23-acetate, 11-deoxy-alisol B, and 11-deoxy-alisol B 23-acetate; their structure is shown in Figure 1) in IR C2C12 cells.

## 2. Materials and Methods

### 2.1. ART Preparation

**Figure 2:**
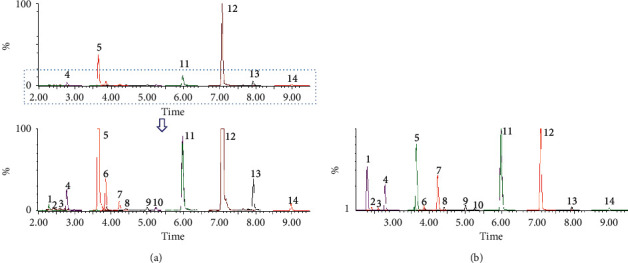
UPLC–Q-TOF–MS chromatography of TTE (a) and reference substances (b); (1) 16-oxo-alisol A (2) 16-oxo-alisol A 23-acetate, (3) 16-oxo-alisol A 24-acetate, (4) alisol C (5) alisol C 23-acetate, (6) alisol (L) (7) alisol A (8) alisol A 23-acetate, (9) alisol A 24-acetate, (10) alisol L 23-acetate, (11) alisol B (12) alisol B 23-acetate, (13) 11-deoxy-alisol B (14) 11-deoxy-alisol B 23-acetate.

According to our previous study [35], the extraction and purification method of Alismatis Rhizoma Triterpenes was as follows. Dried rhizomes of Alismatis Rhizoma were ground into powder (24 mesh) and then twice extracted by a decoction with 80% ethanol for 1 h. The filtrate was concentrated and subjected to chromatography on an HP20 macroreticular resin (Beijing Greenherbs Science and Technology Co., Beijing, China) column by using deionized water, 40% ethanol, and 75% ethanol as eluent. After collecting and concentrating the 75% ethanol fraction, the ART yield was determined to be 0.72%. An ACQUITY UHPLC I-Class system coupled with a Xevo XS quadrupole time of flight mass spectrometer (Waters, Milford, MA, USA) was used, and the LC conditions were based on a previous LC-Q-TOF-MS method [12, 36]. Chromatographic separation was carried out at 40°C on a Waters CORTECS C18 column (2.1 mm × 100 mm; 1.6 *μ*m), with 0.1% of formic acid in water as mobile phase A and acetonitrile B as mobile phase B. Gradient elution was performed as follows: 45%–45% B for 0–0.5 min, 46%–65% B for 0.5–2 min, 65%–90% B for 2–7 min, and 90%–100% B at 7–10 min. The flow rate was 0.25 mL/min. The mass-spectrometry conditions were optimized as follows: dissolvent gas temperature, 500°C; capillary voltage, 3.5 kV; source temperature, 150°C; dissolvent gas flow, 800 L/h; and cone gas flow, 50 L/h. The MS scan range was m/z 50–1000, and the collision energy was set at 20 eV. For quantitation of ART, standard and sample solutions were prepared as follows: a series of working standard solutions of fourteen analytes [(1) 16-oxo-alisol A, (2) 16-oxo-alisol A 23-acetate, (3) 16-oxo-alisol A 24-acetate, (4) alisol C, (5) alisol C 23-acetate, (6) alisol L, (7) alisol A, (8) alisol A 23-acetate, (9) alisol A 24-acetate, (10) alisol L 23-acetate, (11) alisol B, (12) alisol B 23-acetate, (13) 11-deoxy-alisol B, (14) 11-deoxy-alisol B 23-acetate] were freshly prepared by diluting the mixed standard solutions with acetonitrile at the ratios of 2, 5, 10, 20, 50, 100, 200, 500, 1000, and 2000 ng/mL (Table S1). The ART samples: 20 mg powders were dissolved with 50 mL acetonitrile, then centrifuged at 12000 rpm for 10 min. The supernatant was diluted 100 times with acetonitrile to obtain a sample solution. Sample and working standard solutions were injected into LC-MS to acquire peak area responses (the detailed quantitative ion channel was shown in Table S2). Chromatogram of the sample (a) and standard solutions (b) were shown in Figure 2. According to the working standard concentration linearity curve (Table S1), fourteen triterpenes in ART were detected. ART containing fourteen triterpenes amounted to 885.9 mg/g (Table 1).

The ingredients of ARTC were obtained according to our previous research [37]. Then, ARTC was prepared as follows: fourteen pure triterpenes were accurately weighed and mixed: 16-oxo-alisol A (1.42 mg), 16-oxo-alisol A 23-acetate (0.73 mg), 16-oxo-alisol A 24-acetate (0.75 mg), alisol C (3.52 mg), alisol C 23-acetate (18.84 mg), alisol L (3.26 mg), alisol A (2.86 mg), alisol A 23-acetate (0.94 mg), alisol A 24-acetate (1.12 mg), alisol L 23-acetate (1.01 mg), alisol B (13.27 mg), alisol B 23-acetate (32.26 mg), 11-deoxy alisol B (6.57 mg), and 11-deoxy alisol B 23-acetate (2.04 mg). The contents of these compounds in ART extract and ARTC were equal. In *in vitro* experiment, the mother liquid of ARTC (100 mg/mL in DMSO) was then diluted with a DMEM medium to the required drug concentrations and filtered with a 0.22 *μ*m Millipore filtration system (Lot. No. R9BA69583) to eliminate bacteria.

### 2.2. Animal

Ethical approval for the present study was obtained from the Ethical Committee of the Fujian Medical University, China. Male C57BL/6J mice (6 weeks old and weighing 16–18 g) were purchased from Shanghai SLAC Company. The production license number was SCXK (Shanghai) 2017-0005. Mice were fed in the Fujian Medical University SPF Animal Center, and the breeding facilities used permission license number SYXK (Fujian) 2016-0006. The temperature was 22–24°C, the relative humidity was 50%–70%, and the light/dark cycle was 12/12 h. All animal experiments were performed in accordance with the Guidelines for the Care and Use of Laboratory Animals of Fujian Medical University (Fujian, China).

### 2.3. Construction of IR-Mouse Model

The male C57BL/6J mice were randomly divided into Chow (*n* = 10) and model (*n* = 30) groups. The Chow group was given a chow-fat diet, and the model group was given HFD. The Chow-fat diet (CHOW, D12450B, 10% calories from fat) and HFD (D12492, 60% calories from fat) were purchased from Guangdong Animal Center (Guangzhou, China). After 10 weeks, the mice were fasted for 12 h and then fasting blood glucose (FBG), fasting insulin (FINS), and IPITT were measured. Homeostasis model assessment of IR index (HOMA-IR) was also calculated to determine whether the IR model was established successfully (Figure S1).

The HOMA-IR formula was as follows:

(1) $$\begin{array}{c} \text{HOMA}-\text{IR}=\frac{\left[\text{FBG}\left(\text{mmol}/\text{L}\right)*\mathrm{FINS}\left(\mathrm{mIU}/L\right)\right]}{22.5}. \end{array}$$

### 2.4. Mouse Grouping and Administration

After establishing the IR-mouse model, the model group was randomly divided into three groups, namely, a positive control group (HFD + POS), an HFD group, and an HFD + ART group. Each group had 10 mice whose weights were recorded. The CHOW group was given a standard diet, the HFD + POS group was given HFD added with 200 mg/kg/d metformin, and the HFD + ART group was given HFD added with 200 mg/kg/d ART. All drugs were mixed as feed supplements through the equivalent incremental method and fed for 4 weeks. Positive drug metformin was bought from Farmhispania (Farmhispania S.A. Barcelona, Spain).

### 2.5. Intraperitoneal Glucose Tolerance Tests (IPGTT) and Intraperitoneal Insulin Tolerance Test (IPITT)

For IPGTT tests, five mice per group were randomly selected, fasted for 12 h, and intraperitoneally injected with 2 g/kg glucose solution. For IPITT tests, five mice per group were randomly selected, fasted for 6 h, and intraperitoneally injected with 0.5 U/kg insulin solution. A glucometer was used to measure FBG and BG 15, 30, 60, and 120 min after intraperitoneal injection of each mice. The AUC in blood sugar was calculated as follows:

(2) $$\begin{array}{c} \text{AUC}=\left(\text{FBG }+\;15\text{ min BG}\right)\times \frac{15}{2}+\left(15\text{ min BG }+\;30\text{ min BG}\right)+\frac{15}{2}+\left(30\text{ min BG }+\;60\text{ min BG}\right)+\frac{30}{2}+\left(60\text{ min BG }+\;120\text{ min BG}\right)\times \frac{60}{2}. \end{array}$$

### 2.6. Western Blot Assay

Total protein was extracted with RIPA lysis buffer added with protease inhibitor (Beyotime Biotechnology, China) on ice, and the concentration of protein samples was determined by the BCA method. Cell-membrane proteins were extracted using a Mem-PERa Plus Membrane Protein Extraction Kit according to the manufacturer's protocol. Samples were separated with 10% SDS/PAGE gel and then transferred onto NC membrane (Merck Millipore, Bedford, MA, USA). The protein band was blocked with 5% skim milk for 1 h at room temperature and incubated with primary antibodies overnight at 4°C. The primary antibodies included the following: AMPK, p-AMPK, GLUT-4, AKT, P-IRS-1 (ser307), IRS-1 (ser307), P-AKT, and antiactin mouse (purchased from Santa Cruz, USA); and JNK, P-JNK, and PI3K (purchased from Abcam, USA). The information of all antibodies is shown in Table S3. After washing with TBST buffer, bands were incubated with secondary antibodies. HRP AffiniPure Goat Anti-Mouse lgG (H + L) and HRP AffiniPure Goat Anti-Rabbit lgG (H + L) were purchased from Emarbio Science & Technology (Beijing, China). Proteins were detected and visualized using an ECL chemiluminescence kit (Jiancheng-BIO, Nanjing China). Actin served as an internal reference. The expressed proteins were quantified by densitometry analysis by using IMAGEJ software (National Institutes of Health, Bethesda, MD, USA).

### 2.7. Cell Culture and Treatment

C2C12 cells were cultured in DMEM media (with 10% FBS and 100 U/mL penicillin, and 100 mg/mL streptomycin) in a 5% CO_2_ incubator at 37°C. When cell density reached 70%–80%, the culture media was changed into high-glucose DMEM with 2% horse serum (HS) to induce cellular differentiation. To establish the C2C12 cell IR model, C2C12 cells were treated with 0.5 mM PA for 16 h. To evaluate the therapeutic potential of ART in the IR cell model, C2C12 cells were placed onto six-well plates (0.25 × 10^6^/well), and cellular differentiation was induced with 2% HS. Hunger treatment with low-glucose DMEM (1% BSA) was conducted for 12 h, and then grouping and administration were conducted as follows:

  Control group (Con): 2% HS DMEM + 0 mM PA;
  Model group (PA): 2% HS DMEM + 0.5 mM PA;
  PA + ARTC25: 2% HS DMEM + 0.5 mM PA + 25 *μ*g/mL ARTC;
  PA + ARTC50: 2% HS DMEM + 0.5 mM PA + 50 *μ*g/mL ARTC;
  PA + ARTC100: 2% HS DMEM + 0.5 mM PA + 100 *μ*g/mL ARTC.

After intervention for 16 h, the culture media (with/without 100 nM insulin) was changed and incubated for 30 min.

### 2.8. MTT Assay

Differential C2C12 cells were seeded onto 96-well plates with low-glucose DMEM culture medium (1% BSA). After 0.5 mM PA treatment for 16 h, the medium was changed with 2% HS and incubated with high-glucose DMEM (added with different final concentrations of ARTC, namely, 0, 12.5, 25, 50, 100, 200, 300, and 400 *μ*g/mL). After culturing for 24 h, the culture media was absorbed and washed with PBS twice. About 100 *μ*L of 0.5 mg/mL MTT solution was added, and the cells were cultured in a 37°C incubator for 4 h in darkness. Then, the MTT reagent in each well was removed and DMSO (100 *μ*L/well) was added. The mixture was vortexed and OD_570 nm_ was measured. To examine the influence of PA on C2C12 cell viability, we used the same method as above with the PA final concentrations set as 0, 0.1, 0.2, 0.3, 0.4, 0.5, 0.7, and 1 mM.

Cell viability was calculated as follows:

(3) $$\begin{array}{c} \text{Cell viability}\left(\text{\%}\right)=\frac{\left({\text{OD}}_{\text{treatment}}-OD_{\text{blank}}\right)}{\left({\text{OD}}_{\text{control}}-OD_{\text{blank}}\right)}\times 100\text{\%}. \end{array}$$

### 2.9. Glucose-Uptake Assay

The glucose-uptake rate was detected by the fluorescent d-glucose analog 2-[N-(7-nitrobenz-2-oxa-1, 3-diazol-4-yl) amino]-2-deoxy-d-glucose method. After cell differentiation by HS for 4 days, cells were seeded onto 24-well plates (incubated with or without insulin (100 nM) for 15 min and treated with 50 *μ*M 2-NBDG for 20 min). All procedures were conducted following the instructions of the Screen QuestTM Fluorimetric Glucose Uptake Assay Kit (AAT Bioquest, CA, USA). Fluorescence intensity was detected by fluorescent spectrometry (BIO-TEK, USA) at an excitation of 485 nm and an emission of 535 nm.

### 2.10. Statistical Analysis

All data were analyzed using SPSS version 17.0 software (SPSS Inc., Chicago, IL, USA). Results are expressed as the mean ± SEM. All data were analyzed by one-way ANOVA, and Student's *t*-test was subsequently conducted for multiple comparisons. *P* < 0.05 was considered as a significant difference between each group.

## 3. Results

### 3.1. Determination of Triterpenes in ART

UPLC–Q-TOF–MS analysis showed that 16-oxo-alisol A, 16-oxo-alisol A 23-acetate, 16-oxo-alisol A 24-acetate, alisol C, alisol C 23-acetate, alisol L, alisol A, alisol A 23-acetate, alisol A 24-acetate, alisol L 23-acetate, alisol B, alisol B 23-acetate, 11-deoxy-alisol B, and 11-deoxy-alisol B 23-acetate were the major triterpenes in ART. The number of total triterpenoids containing 14 triterpenes in ART was 885.9 mg/g (Table 1).

### 3.2. ART Treatment Improves IR by Regulating Body Weight, Glucose Tolerance, HbA1C, FFA, and Inflammatory Factors in IR Mice

**Figure 3:**
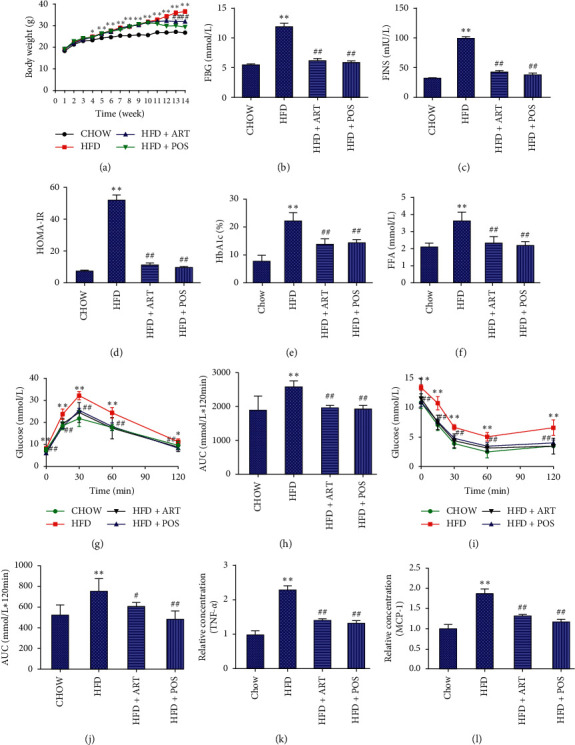
ART treatment improves IR by regulating glucose tolerance and insulin sensitivity in IR mice. (a) Body weight of four groups of mice, namely, the CHOW group (normal diet, *n* = 10), HFD group (high-fat diet, *n* = 10), HFD + ART group (high-fat diet with ART treatment, 200 mg/kg, *n* = 10), and HFD + POS group (high-fat diet with positive drug metformin treatment, 200 mg/kg, *n* = 10), in 14 weeks were recorded. (b–f) Result of FBG (b), FIINS (c), HOMA-IR (d), HbA1C (e), and FFA (f), level in the blood of each group. (g and h) The glucose level (g) of each group in the IPGTT assay was determined, and the AUC (h) was calculated. Glucose level (i) of each group in the IPITT assay was determined, and the AUC (j) was determined as well. (k and l) Result of tumor TNF-*α* (k) and MCP-1 (l) expression of each group.  ^*∗∗*^*P* < 0.01 vs. CHOW group, ##*P* < 0.01 vs. HFD group.

A high-fat diet-induced IR model (HFD) was established following our previous studies [11, 38] to explore the ART therapeutic potential in IR mice. We used ART treatment to intervene with these mice and observe the response. At the time point of 10 weeks, the IR model was established (Figure S1). After ART (200 mg/kg) treatment for 4 weeks (11–14 weeks), the body weight of IR mice (HFD + ART group) was significantly decreased compared with that of HFD group mice (Figure 3(a)). Efficacy monitoring indicators were considered. Specifically, the level of FBG, FINS, HbA1c, FFA with a close relationship with IR occurrence and promotes the development of IR-type diabetes [39]) in serum was determined, and the HOMA-IR in each group was conducted. After ART treatment, FBG, FIINS, HbA1C, FFA concentration, and HOMA-IR were decreased compared with those of HFD group mice (Figures 3(b)–3(f)). IPGTT and IPIGG were performed after 4 weeks of ART treatment to investigate the ART effect on glucose. As shown in Figure 3(g), the HFD + ART group exhibited a significantly low level of blood glucose concentrations at 0, 15, 30, 60, and 120 min in IPGTT assay compared with HFD group mice. The area under the curve (AUC) of blood glucose concentrations was also significantly decreased in the HFD + ART group than in the HFD group (*P* < 0.01) (Figure 3(h)). In the IPITT assay, HFD + ART group showed more significant and faster glucose reduction than the HFD group at 0, 15, 30, 60, and 120 min (*P* < 0.01) (Figure 3(i)). The AUC of blood glucose concentrations at HFD + ART group was lower than that at HFD group (*P* < 0.05) (Figure 3(j)). These results indicated that ART treatment could promote glucose metabolism. Meanwhile, the HFD-induced upregulation of serum tumor TNF-*α* and MCP-1 expression was also reduced by ART treatment (Figures 3(k) and 3(l)).

### 3.3. ART Treatment Regulates IR-Associated Protein Expression

**Figure 4:**
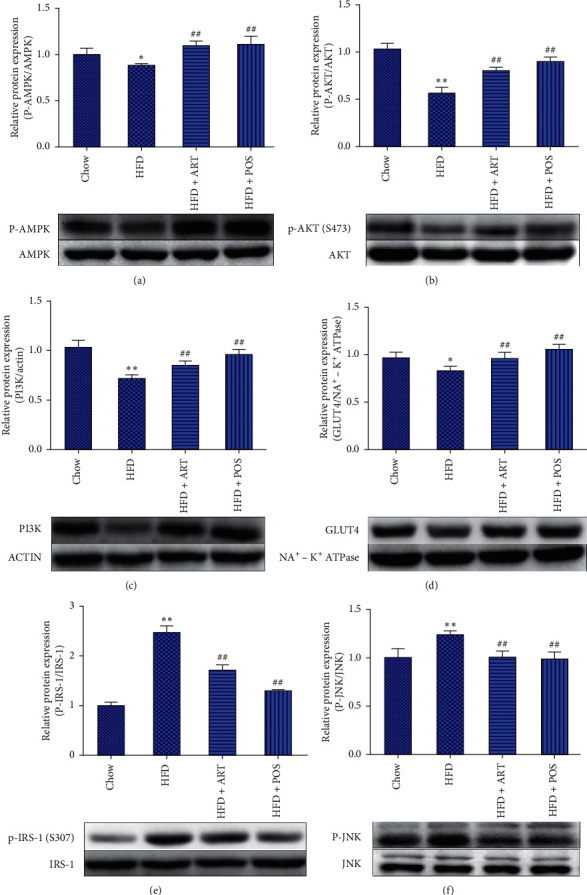
ART treatment could regulate IR-associated gene expression in IR mice. (a) p-AMPK, relative protein expression of each group. (b) p-AKT relative protein expression of each group. (c) PI3K relative protein expression of each group. (d) GLUT4 relative protein expression of each group. (e) p-IRS-1 relative protein expression of each group. (f) p-JNK relative protein expression of each group.  ^*∗*^*P* < 0.05 compared with the Chow group,  ^*∗∗*^*P* < 0.01 compared with the Chow group, #*P* < 0.05 compared with the HFD group, ##*P* < 0.01 compared with the HFD group.

We analyzed IR-associated protein expression in the skeletal muscle of mice using Western blot analysis to evaluate the evidence base of ART treatment of IR. As shown in Figure 4, HFD group mice showed significantly downregulated AMPK (phosphorylated, Figure 4(a)), AKT (phosphorylated, Figure 4(b)), PI3K (Figure 4(c)), and GLUT4 (Figure 4(d)) compared with CHOW group. Moreover, HFD group mice showed significantly upregulated relative protein expression of IRS-1 (phosphorylated, Figure 4(e)) and JNK (phosphorylated, Figure 4(f)) compared with CHOW. Interestingly, ART treatment could reverse all IR-associated gene dysregulations (Figure 4). The relative expression levels of p-AMPK, p-AKT, p-IRS-1, and p-JNK protein were quantified by gray analysis and normalized to corresponding nonphosphorylated proteins. The relative expression levels of PI3K protein were quantified by gray analysis and normalized to actin. The relative expression levels of GLUT4 protein were quantified by gray analysis and normalized to Na^+^ − K^+^-ATPase. All results were represented as the mean ± SEM. ART treatment could regulate IR-associated gene expression in the skeletal muscle of high-fat diet-induced IR mice via AMPK/JNK and IRS-1/PI3K/AKT/GLUT4 signaling pathway.

### 3.4. ARTC Ameliorates Glucose Consumption and IR-Associated Inflammation in Skeletal Muscle Cells

**Figure 5:**
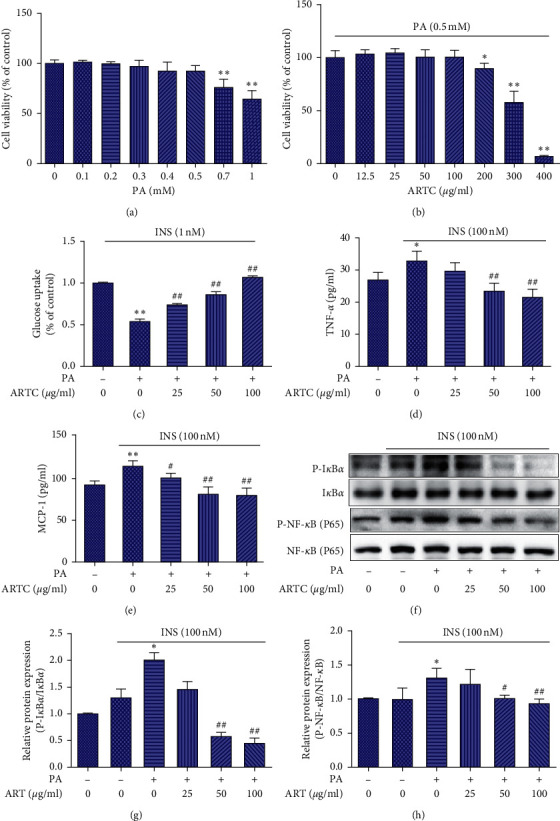
ARTC treatment in PA-induced IR C2C12 cells. (a) Cell viability was determined after 24 h PA treatment by MTT assay. (b) Cell viability in different concentrations of ARTC (0, 12.5, 25, 50, 100, 200, 300, and 400 *μ*g/ml) was measured by MTT assay. (c) Glucose uptake was detected in ARTC-treated IR C2C12 cells. (d) The concentration of proinflammatory cytokines TNF-*α* by ARTC treatment. (e) The concentration of proinflammatory cytokines MCP-1 by ARTC treatment. (f) Western blotting. (g) p-*IκBα* relative protein expression of each group. (h) p-NF-*κB* relative protein expression of each group. #*P* < 0.05, ##*P* < 0.01 vs. IR model group;  ^*∗*^*P* < 0.01,  ^*∗∗*^*P* < 0.05 vs. control group, *n* = 3.

Cell viability in response to different PA concentrations (0, 0.1, 0.2, 0.3, 0.4, 0.5, 0.7, and 1 mM) and ARTC concentrations (0, 12.5, 25, 50, 100, 200, 300, and 400 *μ*g/ml) was measured after treatment for 24 h (Figures 5(a) and 5(b)) to test the cytotoxicity of PA and ARTC. As a result, PA at 0.5 mM reduced cell viability to approximately 85% of control, and 25–100 *μ*g/ml ARTC doses were chosen to further explore the improvement effect in IR cell. PA-induced IR C2C12 cell model was constructed [40]. After 16 hours of PA intervention on C2C12 cells, the glucose consumption of the cells decreased by 12.95% (*P* < 0.01) compared with that of the control group. In this model, ARTC treatment could reverse the PA-induced decrease in glucose uptake in a dose-dependent manner (Figure 5(c)). Meanwhile, the levels of proinflammatory cytokines TNF-*α* and MCP-1, which reflect inflammation status in IR C2C12 cells, significantly decreased by ARTC treatment than those of IR C2C12 cell model group (*P* < 0.05, Figures 5(d) and 5(e)). Western blot analyses of p-*IκBα* and p-NF-*κB* (Figures 5(f)–5(h)) were performed for further confirmation. The results showed that ARTC had an anti-inflammatory effect on IR C2C12 cells.

### 3.5. ARTC Treatment Could Regulate IR-Associated Protein Expression in PA-Induced IR C2C12 Cells

**Figure 6:**
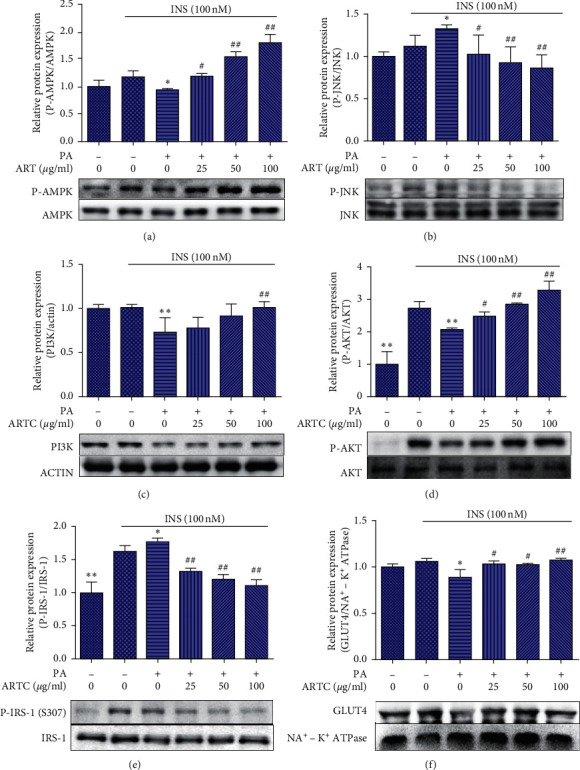
Effects of ARTC on the protein expression of IR-associated genes related to the AMPK/JNK and IRS-1/PI3K/AKT/GLUT4 signaling pathway in PA-induced IR C2C12 cells. (a) Western blot analysis of p-AMPK expression in each group. (b) Western blot analysis of p-JNK expression in each group. (c) Western blot analysis of p-IRIS-1 expression in each group. (d) Western blot analysis of p-IRIS-1 expression in each group. (e) Western blot analysis of PI3K expression in each group. (f) Western blot analysis of GLUT4 expression in each group. #*P* < 0.05, ##*P* < 0.01 vs. model group;  ^*∗*^*P* < 0.01,  ^*∗∗*^*P* < 0.05 vs. control group, *n* = 3.

Additional comprehensive protein detection was performed to further confirm the ARTC regulation on IR-associated genes. In PA-induced IR cells, the protein expression levels of p-AMPK, p-JNK PI3K, p-AKT, and GLUT4 were suppressed. By contrast, the level of p-IRIS-1 increased significantly. As shown in Figure 6, the expression levels of p-AMPK (Figure 6(a)), p-JNK (Figure 6(b)) PI3K (Figure 6(c)), p-AKT (Figure 6(d)), p-IRIS-1 (Figure 6(e)), and GLUT4 (Figure 6(f)) could be reversed after ARTC intervention. Furthermore, the relationship was dose dependent. The relative expression levels of p-AMPK, p-AKT, p-IRS-1, and p-JNK protein were quantified by gray analysis and normalized to corresponding nonphosphorylated proteins. The relative expression levels of PI3K protein were quantified by gray analysis and normalized to actin. The relative expression levels of GLUT4 protein were quantified by gray analysis and normalized to Na^+^ − K^+^-ATPase. All results were represented as the mean ± SEM. These results indicated that ARTC treatment had a significant regulatory effect on the expression of IR-associated genes related to AMPK/JNK and IRS-1/PI3K/AKT/GLUT4 signaling pathway in C2C12 cells induced by PA.

## 4. Discussion

IR is a complex metabolic syndrome that may occur with obesity, diabetes, or cardiovascular diseases and lead to many abnormalities [41]. In a mouse model, IR has manifested as hyperglycemia, hyperinsulinemia, increased HOMA-IR, impaired glucose tolerance and insulin tolerance, dysglycemia, and inflammatory factor disorder [42–44]. At the cellular level, IR primarily manifests as glucose metabolism disorder caused by insulin stimulation to target cells or tissues, especially skeletal muscle tissue [45]. Glucose metabolism usually refers to the process of glucose absorption and utilization by organisms and includes mostly AMPK/JNK and IRS-1/PI3K/AKT/GLUT4-mediated insulin signaling pathway disorder.

Currently recognized IR-mouse models include transgenic, knockout, genetic inherited (spontaneous), and inducible IR [46]. Owing to their stability and convenience, male C57BL/6J mice fed with HFD for 10 weeks were adopted as the IR-mouse model [11, 38]. To determine the actual material foundation of the anti-IR effect of ART from AR, UPLC-Q-TOF-MS was used to identify and determine the major ingredients from ART. To evaluate the anti-IR effect *in vitro*, we used the ARTC (a mixture of 16-oxo-alisol A, 16-oxo-alisol A 23-acetate, 16-oxo-alisol A 24-acetate, alisol C, alisol C 23-acetate, alisol L, alisol A, alisol A 23-acetate, alisol A 24-acetate, alisol L 23-acetate, alisol B, alisol B 23-acetate, 11-deoxy-alisol B, and 11-deoxy-alisol B 23-acetate, whose contents were equal to that of the ART extract).

In order to prove triterpenes are medicinal ingredients that alleviate high-fat diet-induced insulin resistance in skeletal muscle of mice, both *in vivo* and *in vitro* experiments were conducted. In vivo, as for AR triterpenes extract (ART) preparation, the ethanol extraction process and macroporous adsorption resin purify process of AR was used. As a result, the ART containing 14 structures clearly triterpenes amounted to 885.9 mg/g (the purity of triterpenes in ART has reached 88.59%). *In vitro*, in order to further confirm that triterpenes are the medicinal ingredients, ART component compatibility (ARTC, the composition and quantity of triterpenes equivalent to the ART extract triterpenes) was used for palmitate-treated IR C2C12 cells to exclude other components (such as sesquiterpenoids). So, ARTC preparation: fourteen pure triterpenes were accurately weighed and mixed: 16-oxo-alisol A (1.42 mg), 16-oxo-alisol A 23-acetate (0.73 mg), 16-oxo-alisol A 24-acetate (0.75 mg), alisol C (3.52 mg), alisol C 23-acetate (18.84 mg), alisol L (3.26 mg), alisol A (2.86 mg), alisol A 23-acetate (0.94 mg), alisol A 24-acetate (1.12 mg), alisol L 23-acetate (1.01 mg), alisol B (13.27 mg), alisol B 23-acetate (32.26 mg), 11-deoxy alisol B (6.57 mg), and 11-deoxy alisol B 23-acetate (2.04 mg); it only contained these 14 triterpenes rather than other components.

So, we investigated the anti-IR therapeutic effect of ART in an IR-mouse model and a C2C12 cell model. First, the IR-mouse model fed with HFD for 10 weeks was successfully constructed. Through FBG level measurement, IPGTT assay, and HOMA-IR score calculation, we found that the HFD group showed significant IR characters, indicating that the IR-mouse model was established. Then, after ART therapy for 4 weeks, the body weight significantly decreased. Through IPGTT and IPIGG assays, we found that ART could improve IR. Meanwhile, HbA1c as a standard marker for glycemic excursion in diabetic patients was also identified as an IR-prediction marker [47]. Notably, IR is a disease with complex networks of glucose metabolism and fat metabolism. When the tissue is insulin sensitive, excessive FFA in circulation creates IR [48]. Afterwards, the increase in lipolysis produces and releases more FFA, which forms the inflammatory basis in IR disease [49]. Herein, HbA1c, FFA, TNF-*α*, and MCP-1 level in the ART treatment group all significantly decreased compared with the HFD group. Moreover, considering the critical role of skeletal muscle in insulin-stimulated systemic glucose metabolism, skeletal muscle glucose metabolism disorders could affect systemic glucose homeostasis and insulin sensitivity [50, 51]. Skeletal muscle is an important tissue for the body's glucose uptake and utilization and plays a major role in maintaining the dynamic balance of blood glucose. Accordingly, we detected IR-associated gene expression in mouse skeletal muscle and found that ART treatment regulated the AMPK/JNK and IRS-1/PI3K/AKT/GLUT4 signaling pathway. AMPK also ameliorated inflammation and maintained energy homeostasis by inhibiting NF-*κB* activity. IR induced by HFD activated JNK, which led to decreased phosphorylation of IRS-1 tyrosine residues and prevented the transfer of GLUT4 from the intracellular storage to the plasma membrane through the IRS-1/PI3K/Akt signaling cascades. IRS-1/PI3K/Akt signaling pathways are the classical pathways in the regulation of glucose uptake and metabolism [14, 24–29]. PI3K becomes the focus on the effects of cell growth and cell proliferation by nutrition, especially glucose uptake and cell-cycle regulation. AKT is involved in the signaling pathway of glucose-stimulated insulin secretion in skeletal muscle cells as signaling molecules in insulin secretion, the effects of translocation of GLUT4 in skeletal muscle [52, 53]. The above research shows that the AMPK/JNK and IRS-1/PI3K/AKT/GLUT4 signaling pathway is closely implicated in HFD-induced IR. Previous studies have suggested that in HFD-induced hyperlipidemia mouse, the methanol extract of the tuber of *Alisma orientale* can lower serum lipid levels and prevents hepatic steatosis pathogenesis by inhibiting the expression of hepatic lipogenic genes [7, 54]. Triterpenes isolated from AR have the activity of promoting glucose uptake [55]. Recent studies have also shown that alisol A-24-acetate promotes glucose uptake via the activation of AMPK, phosphorylation of JNK and p38 in C2C12 myotubes. [56]. Alisol B 23-acetate protects against nonalcoholic steatohepatitis in mice via farnesoid X receptor activation [57]. Furthermore, the NF-*κB* signaling pathway is obviously suppressed in liver cells treated with alisol F and 25-anhydroalisol F [58]. Alisol A, alisol C, alisol B 23-acetate, alisol A-24-acetate, alisol C-23-acetate, and 16-oxo-alisolA have been described with modulating effects of glucose uptake [13]. So, compared to these triterpene compounds, the ART is characterized by multiple triterpenes components and may show better activity in improving the IR than a single component, different triterpenes in AR have different sets of gene targets [5]. So, the overall effect of all triterpenes may be better than the pure one triterpene.

Although AR compounds have been found to improve genes related to glucose and lipid metabolism, the regulation of the insulin signaling pathway has not yet been systematically reported. To further facilitate evidence-based cellular exploration, the IR model of C2C12 cells induced by PA was accustomed. After the ARTC intervention, we found that ARTC treatment promoted PA-induced glucose consumption in a dose-dependent manner [39]. The intervention of ART reduced the expression of TNF-*α* and MCP-1 levels in the IR model cells, implying the anti-inflammatory effect of ART. It also led to the activation of AMPK and AKT and the inhibition of *IκBα*, NF-*κB*, JNK, and IRS-1, ultimately promoting GLUT4 expression. In summary, ARTC exerted a significant regulatory effect on IR-associated genes related to the AMPK/JNK and IRS-1/PI3K/AKT/GLUT4 signaling pathway in C2C12 cells induced by PA.

## 5. Conclusions

The above results showed that ART attenuated the impairment of AMPK/JNK and IRS-1/PI3K/AKT/GLUT4-mediated insulin signaling in skeletal muscles of HFD-fed mice. ART treatment significantly ameliorated HFD-induced weight gain and improved glucose tolerance in mice. The expression levels of proinflammatory cytokines were markedly attenuated by ART in various in vivo models. ART treatment markedly augmented AMPK phosphorylation expression in the skeletal muscle of mice. Furthermore, the intervention of ARTC reduced the expression levels of TNF-*α* and MCP-1 in IR model cells, leading to the activation of AMPK and AKT/PI3K and the inhibition of *IκBα*, NF-*κB*, JNK, and IRS-1, ultimately promoting GLUT4 expression. In conclusion, our results demonstrated that ART markedly ameliorated IR in vivo and *in vitro*. ART exerted a notable alleviated IR effect by regulating IR-associated gene expression, and triterpenes were the material basis for the alleviation of IR activity by AR.

## Figures and Tables

**Table 1:** Characterization of 14 triterpenes in ART by UPLC–Q-TOF–MS.

No.	*t*_*R*_	Molecular ion	Error	Fragment ions in positive mode	Molecular	Identity	Contents
	(min)	MS^1^	(ppm)	MS^2^	formula		(mg/g)
1	2.29	505.3536 [M + H]^+^	1.38	487 [M + H − H_2_O]^+^, 469 [M + H − 2H_2_O]^+^, 415 [M + H − C_4_H_10_O_2_]^+^, 397 [M + H − C_4_H_10_O_2_ − H_2_O]^+^	C_30_H_48_O_6_	16-oxo-alisol A	14.2
2	2.41	547.3640 [M + H]^+^	0.91	529 [M + H − H_2_O]^+^, 511 [M + H − 2H_2_O]^+^, 487 [M + H − HAc]^+^, 451 [M + H − HAc − 2H_2_O]^+^, 415 [M + H − C_6_H_12_O_3_]^+^	C_32_H_50_O_7_	16-oxo-alisol A 23-acetate	7.3
3	2.59	547.3642 [M + H]^+^	1.27	529 [M + H − H_2_O]^+^, 511 [M + H − 2H_2_O]^+^, 415 [M + H − C_4_H_8_O]^+^, 397 [M + H − H_2_O − C_4_H_8_O]^+^	C_32_H_50_O_7_	16-oxo-alisol A 24-acetate	7.5
4	2.78	487.3426 [M + H]^+^	0.41	469 [M + H − H_2_O]^+^, 451 [M + H − 2H_2_O]^+^, 415 [M + H − C_4_H_10_O_2_]^+^, 397 [M + H − C_4_H_10_O_2_ − H_2_O]^+^	C_30_H_46_O_5_	Alisol C	35.2
5	3.65	529.3534 [M + H]^+^	0.94	511 [M + H–H_2_O]^+^, 469 [M + H − HAc]^+^, 451 [M + H − HAc − H_2_O]^+^, 415 [M + H − C_6_H_10_O_2_]^+^, 397 [M + H − C_6_H_10_O_2_ − H_2_O]^+^	C_32_H_48_O_6_	Alisol C 23-acetate	188.4
6	3.96	469.3324 [M + H]^+^	1.27	451 [M + H − H_2_O]^+^, 397 [M + H − C_4_H_8_O]^+^	C_30_H_44_O_4_	Alisol L	32.6
7	4.24	491.3745 [M + H]^+^	1.62	473 [M + H − H_2_O]^+^, 455 [M + H − 2H_2_O]^+^, 437 [M + H − 3H_2_O]^+^, 383 [M + H − H_2_O − C_4_H_10_O_2_]^+^	C_30_H_50_O_5_	Alisol A	28.6
8	4.41	533.3849 [M + H]^+^	1.31	515 [M + H − H_2_O]^+^, 497 [M + H − 2H_2_O]^+^, 455 [M + H − HAc − H_2_O] ^+^, 383 [M + H − C_6_H_12_O_3_ − H_2_O]^+^	C_32_H_52_O_6_	Alisol A 23-acetate	9.4
9	5.01	533.3850 [M + H]^+^	1.49	515 [M + H − H_2_O]^+^, 497 [M + H − 2H_2_O]^+^, 455 [M + H − HAc − H_2_O] ^+^, 383 [M + H − C_6_H_12_O_3_ − H_2_O]^+^	C_32_H_52_O_6_	Alisol A 24-acetate	11.2
10	5.23	511.3428 [M + H]^+^	0.78	451 [M + H − HAc]^+^, 397 [M + H − C_6_H_10_O_2_]^+^	C_32_H_46_O_5_	Alisol L 23-acetate	10.1
11	5.99	473.3638 [M + H]^+^	1.47	455 [M + H − H_2_O]^+^, 437 [M + H − 2H_2_O]^+^, 383 [M + H–H_2_O − C_4_H_8_O]^+^	C_30_H_48_O_4_	Alisol B	132.7
12	7.08	515.3745 [M + H]^+^	1.55	497 [M + H − H_2_O]^+^, 479 [M + H − 2H_2_O]^+^, 437 [M + H − H_2_O − HAc]^+^, 383 [M + H − H_2_O − C_6_H_10_O_2_]^+^	C_32_H_50_O_5_	Alisol B 23-acetate	322.6
13	7.94	457.3684 [M + H]^+^	0.44	439 [M + H − H_2_O]^+^, 385 [M + H − C_4_H_8_O]^+^, 367 [M + H − C_4_H_8_O − H_2_O]^+^	C_30_H_48_O_3_	11-deoxy alisol B	65.7
14	8.97	499.3795 [M + H]^+^	1.60	439 [M + H − HAc]^+^, 385 [M + H − C_6_H_10_O_2_]^+^	C_32_H_50_O_4_	11-deoxy alisol B 23-acetate	20.4

## Data Availability

The data used to support the findings of this study are available within the article and the supplementary materials.

## Supplementary Materials

Supplementary Materials Table S1: Linearity curve, linear rang, and linear correlation coefficient r of the 14 compounds. Table S2: The detailed quantitative ion channel of 14 triterpenes in ART by UPLC–MS, Table S3: The information of all antibodies. Figure S1: Insulin resistant mouse model was constructed. Figure S2: Supplementary immunoblots for Figure 4a (A), Figure 4b (B), Figure 4c (C), Figure 4d (D), Figure 4e (E), Figure 5f (F). Figure S3: Supplementary immunoblots for Figure 5e and Figure 5f (A), Figure 6a (B), Figure 6b, Figure 6c, Figure 6d and Figure 6e (C), Figure 6f (D).
