# Supplementary material for: Alismatis Rhizoma Triterpenes Alleviate High-Fat Diet-Induced Insulin Resistance in Skeletal Muscle of Mice
Source: Evid Based Complement Alternat Med. 2021 Feb 2;2021:8857687. doi: 10.1155/2021/8857687 (PMC7875633; doi:10.1155/2021/8857687)
Supplement: Supplementary Materials — Table S1: Linearity curve, linear rang, and linear correlation coefficient r of the 14 compounds. Table S2: The detailed quantitative ion channel of 14 triterpenes in ART by UPLC–MS, Table S3: The information of all antibodies. Figure S1: Insulin resistant mouse model was constructed. Figure S2: Supplementary immunoblots for Figure 4a (A), Figure 4b (B), Figure 4c (C), Figure 4d (D), Figure 4e (E), Figure 5f (F). Figure S3: Supplementary immunoblots for Figure 5e and Figure 5f (A), Figure 6a (B), Figure 6b, Figure 6c, Figure 6d and Figure 6e (C), Figure 6f (D). [file 8857687.f1.docx]

**Supplementary data**

*Table S1. Linearity curve, linear rang and linear correlation coefficient r of the 14 compounds.*

| No. | Compound | Linearity curve | Linear rang /(ng·mL-1) | r |
| --- | --- | --- | --- | --- |
| 1 | 16-oxo-alisol A | y = 62.82 x - 0.0872 | 2-2000 | 0.9996 |
| 2 | 16-oxo-alisol A 23-acetate | y = 62.86 x - 0.0081 | 2-2000 | 0.9997 |
| 3 | 16-oxo-alisol A 24-acetate | y =62.24 x - 0.0043 | 2-2000 | 0.9995 |
| 4 | alisol C | y = 62.97 x - 0.0183 | 2-2000 | 0.9996 |
| 5 | alisol C 23-acetate | y = 149.1 x +7.654 | 2-2000 | 0.9944 |
| 6 | alisol L | y = 62.99 x - 0.002 | 2-2000 | 0.9995 |
| 7 | alisol A | y = 42.20 x + 0.2373 | 2-2000 | 0.9997 |
| 8 | alisol A 23-acetate | y = 18.56 x+0.0028 | 2-2000 | 0.9990 |
| 9 | alisol A 24-acetate | y = 17.54 x+0.0039 | 2-2000 | 0.9991 |
| 10 | alisol L 23-acetate | y = 62.90 x - 0.0034 | 2-2000 | 0.9996 |
| 11 | alisol B | y = 25.82 x+0.2414 | 2-2000 | 0.9995 |
| 12 | alisol B 23-acetate | y = 152.7 x+0.8597 | 2-2000 | 0.9992 |
| 13 | 11-deoxy alisol B | y = 65.39 x - 0.0066 | 2-2000 | 0.9994 |
| 14 | 11-deoxy alisol B 23-acetate | y = 65.57 x - 0.0056 | 2-2000 | 0.9994 |

*Table S2. the detail quantitative ion channel of 14* *triterpenes in ART by UPLC–MS*

| **No.** | **t_R_** | **quantitative** | **Molecular** | **Molecular ion** | **Daughter ion** | **Contents** |
| --- | --- | --- | --- | --- | --- | --- |
|  | **(min)** | **compounds** | **formula** | **MS^1^** | **MS^2^** | **(mg/g)** |
| 1 | 2.29 | 16-oxo-alisol A | C_30_H_48_O_6_ | 505.35 | 415 | 14.2 |
| 2 | 2.41 | 16-oxo-alisol A 23-acetate | C_32_H_50_O_7_ | 547.36 | 415 | 7.3 |
| 3 | 2.59 | 16-oxo-alisol A 24-acetate | C_32_H_50_O_7_ | 547.36 | 415 | 7.5 |
| 4 | 2.78 | alisol C | C_30_H_46_O_5_ | 487.34 | 415 | 35.2 |
| 5 | 3.65 | alisol C 23-acetate | C_32_H_48_O_6_ | 529.35 | 451 | 188.4 |
| 6 | 3.96 | alisol L | C_30_H_44_O_4_ | 469.33 | 397 | 32.6 |
| 7 | 4.24 | alisol A | C_30_H_50_O_5_ | 491.37 | 383 | 28.6 |
| 8 | 4.41 | alisol A 23-acetate | C_32_H_52_O_6_ | 533.3849 [M+H]^+^ | 383 | 9.4 |
| 9 | 5.01 | alisol A 24-acetate | C_32_H_52_O_6_ | 533.38 | 383 | 11.2 |
| 10 | 5.23 | alisol L 23-acetate | C_32_H_46_O_5_ | 511.34 | 397 | 10.1 |
| 11 | 5.99 | alisol B | C_30_H_48_O_4_ | 473.36 | 437 | 132.7 |
| 12 | 7.08 | alisol B 23-acetate | C_32_H_50_O_5_ | 515.37 | 437 | 322.6 |
| 13 | 7.94 | 11-deoxy alisol B | C_30_H_48_O_3_ | 457.36 | 367 | 65.7 |
| 14 | 8.97 | 11-deoxy alisol B 23-acetate | C_32_H_50_O_4_ | 499.37 | 439 | 20.4 |

*Table S3. The information of all antibodies*

| Antibodies | Manufactor | SOURCE | IDENTIFIER |
| --- | --- | --- | --- |
| Anti-JNK | Abcam | Rabbit | ab179461 |
| Anti-p-JNK | [Abcam](http://www.baidu.com/link?url=SVgBRa2LXyR1Pajid52t52FEqesDk28pAnTLDO0MuNR_CS-epkmo1OAC8jZeXniW) | Rabbit | Ab124956 |
| Anti-PI3K | [Abcam](http://www.baidu.com/link?url=SVgBRa2LXyR1Pajid52t52FEqesDk28pAnTLDO0MuNR_CS-epkmo1OAC8jZeXniW) | Rabbit | Ab191606 |
| Anti-GLUT4 | Santa Cruz | Rabbit | Sc-7938 |
| Anti-Na^+^-K^+^ATPase | Cell Signaling Technology | Rabbit | 3010s |
| Anti-AMPK | Santa Cruz | Rabbit | Sc-25792 |
| Anti-p-AMPK | Santa Cruz | Rabbit | Sc-33524 |
| Anti-AKT | [Cell Signaling Technology](http://www.baidu.com/link?url=0nuLlStyeteufKxaB-8UHkGN_wBJV7ncOLQXF6ptmN4HM5HsxZYnh2vr7n6QUoeRFkL77dLp-yaVFcuFF3cD7ed6zq8YoadM9Hx6SHw5HOq) | Rabbit | 4685s |
| Anti-p-AKT | [Cell Signaling Technology](http://www.baidu.com/link?url=0nuLlStyeteufKxaB-8UHkGN_wBJV7ncOLQXF6ptmN4HM5HsxZYnh2vr7n6QUoeRFkL77dLp-yaVFcuFF3cD7ed6zq8YoadM9Hx6SHw5HOq) | Rabbit | 4060s |
| Anti-p-IRS-1(ser307), | Abcam | Rabbit | ab5599 |
| Anti-IRS-1(ser307) | Abcam | Rabbit | ab47327 |
| Anti-NF-κB(p-65) | Abcam | Rabbit | Ab-32536 |
| Anti-p-NF-κB(p-65) | Cell Signaling Technology | Rabbit | 3033s |
| Anti-IκBα | Abcam | Rabbit | Ab32518 |
| Anti-p-IκBα | Cell Signaling Technology | Rabbit | 2859s |

***2 The establishment of*** ***Insulin resistant mouse model***

To evaluate the ART therapeutic potential in insulin resistance, the IR mouse model were constructed. CHOW group mice showed well mental state, normal diet, energetic and steady weight gain, while HFD group showing depressed state, low activity, sluggish and increased body weight.

For 10 weeks high-fat diet, we tested the level of fasting food glucose (FBG) ,Fasting Inslin (FIINS), Hb1Ac, Body weight and IPGTT in mouse serum and calculated the HOMA-IR in both CHOW and HFD group. As Figure S1. A, B, C, E and F shown FBG, FINS, Hb1Ac and Body weight level of HFD group was significantly higher than CHOW group (*P*<0.01). With the calculating of HOMA-IR (Figure S1. D), the value of HFD group was significantly higher compared with CHOW group . Additionally, the intraperitoneal glucose tolerance test was performed (Figure S1. G). At 0 min, 15 min, 30 min, 60 min, 120 min the blood glucose level of HFD group mice were all higher than CHOW group (*P*<0.01 or *P*<0.05), and the AUC value (Figure S1. H) showed consistently trend (*P*<0.01). These results demonstrated that the IR mouse model was successfully established.


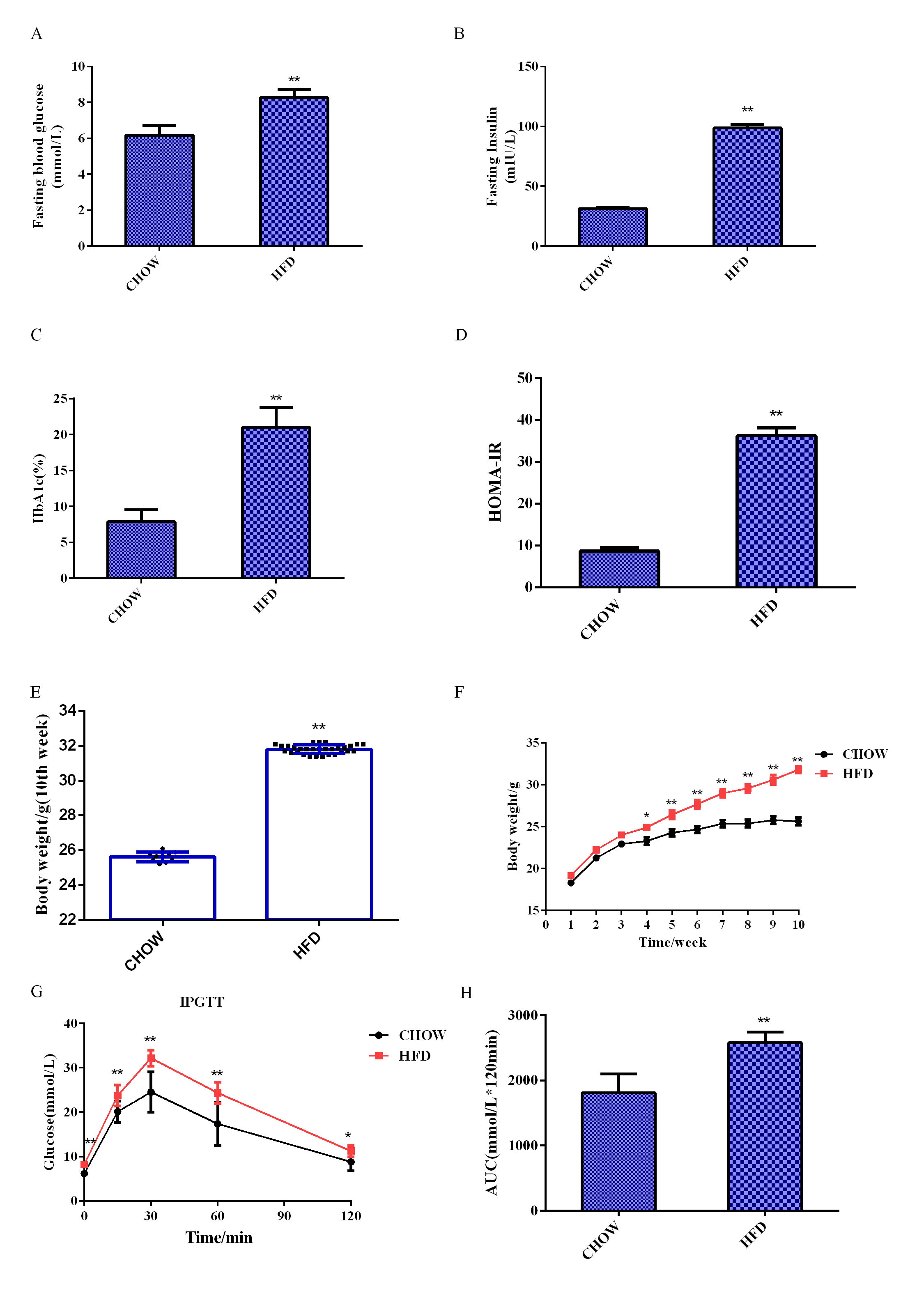


**Figure S1. Insulin resistant mouse model was constructed.** (A, B, C, E) FBG, FINS, Hb1Ac, Body weight of Chow group and high fat diet group (HFD) were detected at 10 weeks treatment. (D) HOMA-IR of each group was calculated with standardized formula (F) Body weight of two groups mice in 10 weeks were recorded, including the CHOW group (chow diet, *n*=10), HFD group (high-fat diet, *n*=30). (G) Blood glucose concentration during the IPGTT assay were measured, and its AUC (H) was shown. Data were the mean ± SD, ***P* < 0.01 *vs* CHOW group.

***3 ART treatment regulates IR-associated gene expression in mice***

**
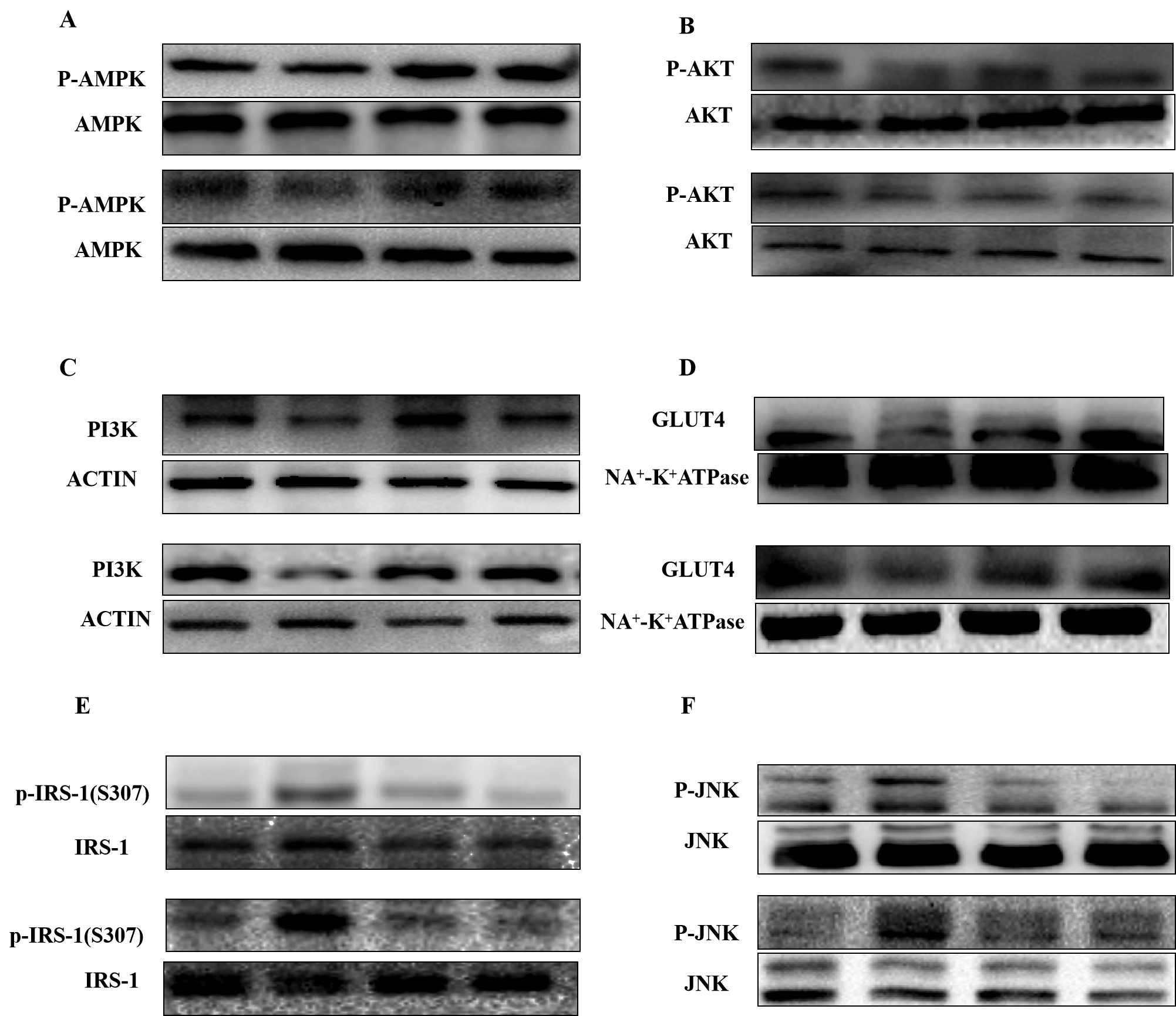
**

Figure S2 | Supplementary immunoblots for Fig. 4a (A), Fig. 4b (B), Fig. 4c (C),

Fig.4d (D), Fig. 4e (E), Fig. 5f (F)

***
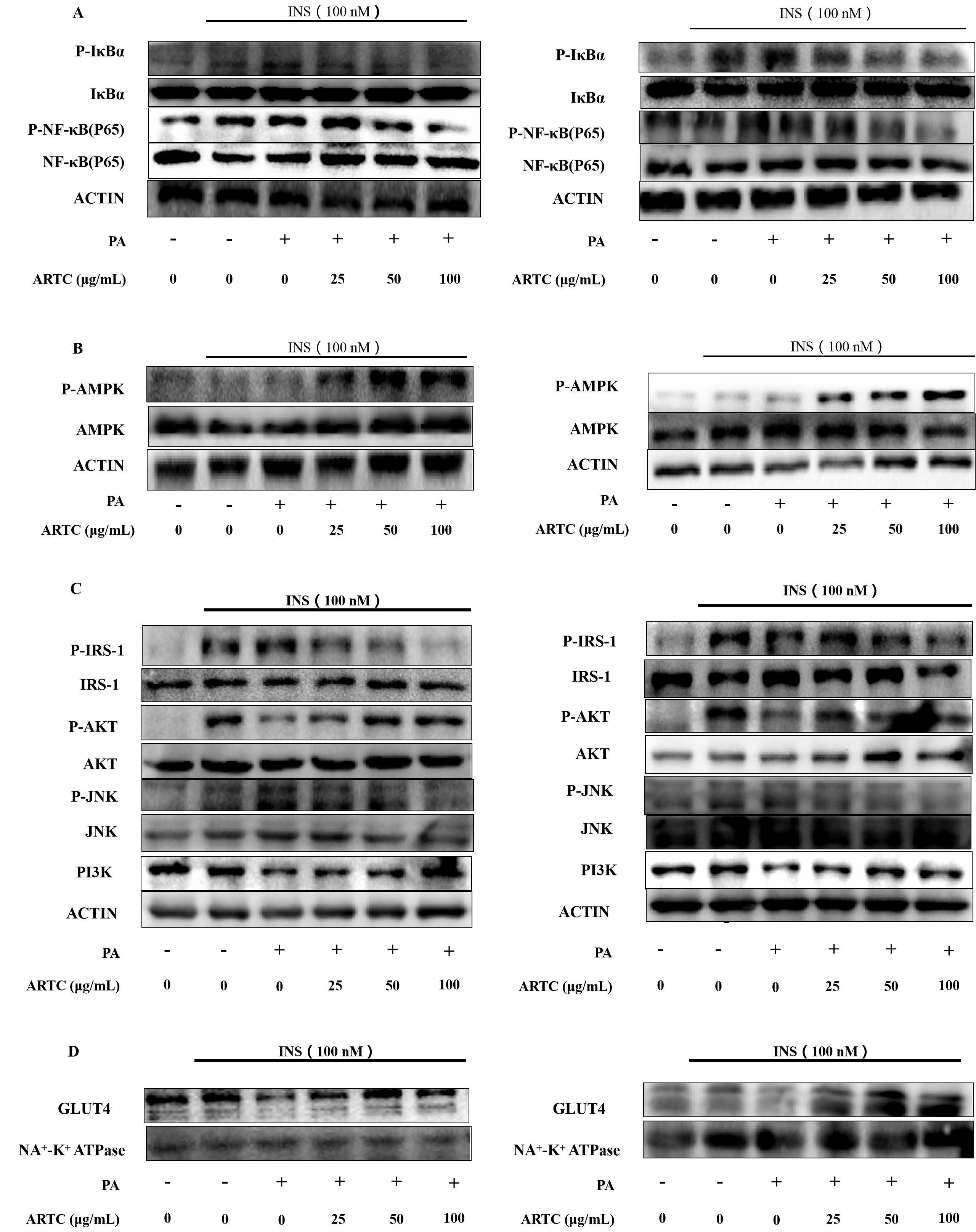
4*** ***ARTC treatment regulates IR-associated gene expression in IR C2C12 cells***

Figure S3. Supplementary immunoblots for Fig. 5e and Fig. 5f (A), Fig. 6a (B), Fig. 6b, Fig. 6c, Fig. 6d and Fig. 6e (C), Fig. 6f (D)
